# Improving Culturally Appropriate Care Using a Community-Based Participatory Research Approach: Evaluation of a Multicomponent Cultural Competency Training Program, Arkansas, 2015–2016

**DOI:** 10.5888/pcd14.170014

**Published:** 2017-08-03

**Authors:** Pearl Anna McElfish, Christopher R. Long, Brett Rowland, Sarah Moore, Ralph Wilmoth, Britni Ayers

**Affiliations:** 1 University of Arkansas for Medical Sciences Northwest, Fayetteville, Arkansas

## Abstract

**Introduction:**

The United States continues to become more racially and ethnically diverse, and racial/ethnic minority communities encounter sociocultural barriers to quality health care, including implicit racial/ethnic bias among health care providers. In response, health care organizations are developing and implementing cultural competency curricula. Using a community-based participatory research (CBPR) approach, we developed and evaluated a cultural competency training program to improve the delivery of culturally appropriate care in Marshallese and Hispanic communities.

**Methods:**

We used a mixed-methods evaluation approach based on the Kirkpatrick model of training evaluation. We collected quantitative evaluation data immediately after each training session (March 19, 2015–November 30, 2016) and qualitative data about implementation at 2 points: immediately after each session and 6 months after training. Individuals and organizational units provided qualitative data.

**Results:**

We delivered 1,250 units of in-person training at 25 organizations. Participants reported high levels of changes in knowledge (91.2%), competence (86.6%), and performance (87.2%) as a result of the cultural competency training. Organizations reported making policy and environmental changes.

**Conclusion:**

Initial outcomes demonstrate the value of developing and implementing cultural competency training programs using a CBPR approach. Additional research is needed to determine the effect on long-term patient outcomes.

## Introduction

The United States continues to grow more racially, ethnically, and culturally diverse (1); approximately 14% of the US population is foreign born (2). By 2044, the United States is projected to become a majority-minority nation, with no one racial or ethnic group expected to represent greater than 50% of the nation’s total population (1). Racially and ethnically diverse communities often encounter sociocultural barriers related to access to quality health care (3,4), such as language barriers (5), access to health insurance (6), lack of culturally competent care (7–10), and implicit bias among health care providers (6,11–13).

Implicit biases often exist outside of conscious awareness and are therefore difficult to acknowledge and remedy (14). The Institute of Medicine found strong evidence of racial bias in health care system policies and in interpersonal interactions (15). Implicit bias may undermine the patient–provider relationship, exacerbating poor health outcomes among racial/ethnic minority populations (11). In response, health care organizations are implementing cultural competency curricula to reduce health care providers’ contributions to inequality (7–10,16,17). The objective of this study was to evaluate a multicomponent cultural competency training program in northwest Arkansas.

## Methods

The population in northwest Arkansas is growing and becoming more racially, ethnically, and culturally diverse. The most dramatic population change in northwest Arkansas is among Hispanic and Pacific Islander populations, which increased approximately 150% and 300%, respectively, from 2000 to 2010 (18–21). Many Hispanic residents are first-generation immigrants, and most Pacific Islanders are migrants from the Republic of the Marshall Islands under the Compact of Free Association (22,23). Northwest Arkansas is home to the largest population of Marshallese in the continental United States (24). Community members and health care providers have voiced concerns about a lack of culturally appropriate care for Hispanic and Marshallese community members.

### Development of the intervention

To better understand the disparities in health between non-Hispanic white populations and racial/ethnic minority populations in northwest Arkansas, the University of Arkansas for Medical Sciences (UAMS) used a community-based participatory research (CBPR) approach during 2 years (January 2013–September 2014) to conduct a needs assessment and set an agenda to address health disparities. The needs assessment consisted of a review of secondary data, community-based surveys, and qualitative interviews. This process is detailed elsewhere (25). The needs assessment revealed culturally and linguistically appropriate care as a major concern in the Hispanic and Marshallese communities.

The needs-assessment team continued to use a CBPR approach to address the communities’ concern for culturally and linguistically appropriate care through the collaborative development, implementation, and evaluation of a cultural competency training series. Partners were the Arkansas Coalition of Marshallese, Gaps in Services to Marshallese Task Force, the local chapter of the League of United Latin American Citizens, Workers’ Justice Center, faith-based leaders, other key stakeholders in the Marshallese and Hispanic communities, and 14 local hospitals and clinics. Using the National Standards for Culturally and Linguistically Appropriate Services in Health and Health Care (26) as a guide, UAMS worked with these partners to identify practice gaps and educational needs. Once practice gaps and educational needs were identified, UAMS worked with partners to outline learning objectives for cultural competency modules (Table 1). Module development was guided by the principle that cultural awareness and appreciation is typically the foundational element of cultural competency (27). Training modules were created and revised collaboratively with Hispanic, Marshallese, and health care partners. Partners worked with UAMS to develop a training curriculum that included relevant, real-life examples that were selected and developed with full stakeholder input. Through curriculum development sessions, stakeholders outlined cultural characteristics that may influence health behaviors. Stakeholders provided insight into common cultural barriers they encountered when accessing health care, as well as common health care beliefs in each culture, the role of family in each culture, and the history of each community in the region.

**Table 1:** Learning Objectives and Corresponding Curricula by Module, Multicomponent Cultural Competency Training Program, Arkansas, 2015–2016

Learning Objective	Curriculum Topics
**General cultural competency training**	
Define cultural competency and relevant concepts	Culture Cultural competency Campinha-Bacote’s model of cultural competency in the delivery of health care (27) Implicit and explicit biases Generalizations Stereotypes
Identify key aspects of cultural competence	Characteristics of culturally competent individuals
Understand the rationale for providing culturally competent care	Legal requirements Ethical requirements Quality of care and health outcomes Bias as a barrier to care
Identify cultural factors that influence health, health behaviors, and response to disease and treatment	Causes and perceptions of illness Attitudes toward treatment and medication adherence Multiple health belief theories
Describe the diversity of northwest Arkansas	Breakdown of ethnic/racial minority groups Washington versus Benton counties Language barriers
Use strategies to overcome barriers to cross-cultural communication	Tips for communicating with patients who have limited English proficiency Best practices for communication through interpreters Cross-cultural communication and interviewing skills
Self-assess own culture, assumptions, stereotypes, and biases	Bias self-assessment (private activity) Cultural competence self-assessment Bridging the gap between providers’ and patients’ beliefs
**Marshallese cultural competency training**	
Describe the background and history of the Marshallese population in the United States and northwest Arkansas	Location of the Republic of the Marshall Islands History and effects of US nuclear testing in the Republic of the Marshall Islands Compact of Free Association Immigration status and disqualification for Medicaid/Medicare Marshallese in northwest Arkansas
Identify health risk factors commonly found in the Marshallese population	Significant health issues Diabetes disparities
Identify cultural characteristics of the Marshallese population that may influence health behaviors	Family structure and roles Importance of religion Nuclear testing and lasting effects on diet and nutrition Exercise and physical activity
Identify common health beliefs in the Marshallese community	Beliefs about causes of illnesses Self-medication, traditional remedies, and healers Less acceptance of Western medicine compared with other populations Lack of preventive care
Use cross-cultural communication skills to appropriately address the health concerns of Marshallese patients	Language as a barrier Tips for effective and appropriate communication Need for Marshallese translators and community health workers
**Hispanic cultural competency training**	
Describe the demographics of the Hispanic population in the United States and northwest Arkansas	National and local statistics Diversity of Hispanic populations Regional differences Generational differences Immigration statuses
Identify health risk factors commonly found in the Hispanic population	Significant health issues Obesity disparities
Identify cultural characteristics of the Hispanic population that may influence health behaviors	Family structure and roles Religion Dietary practices common in northwest Arkansas Differences in physical activity
Identify common health beliefs in the Hispanic community	Beliefs about causes of illnesses Stigma of mental illness Self-medication and herbal remedies Views on US health system
Use cross-cultural communication skills to appropriately address the health concerns of Hispanic patients	Language as a barrier Tips for effective and appropriate communication Need for Spanish translators and community health workers

### Description of the intervention

In December 2014 and January 2015, we developed the cultural competency training (CCT) program by using a multiple module approach, which allowed training sessions to fit the needs of each organization. The general CCT module established the framework for cultural competency, explored the rationale for culturally competent care, examined the issue of implicit bias in health care settings, and discussed knowledge and skills health care providers and workers should possess when working with a diverse patient population. Two additional training modules were developed with emphasis on the Hispanic and Marshallese communities: the Hispanic CCT module and the Marshallese CCT module. The overall goal of the training modules was to contribute knowledge and encourage behavioral changes so that health care professionals and organizations could more effectively serve a diverse patient population.

All cultural competency training modules took place from March 19, 2015, to November 30, 2016, and were 1 hour in length. The general CCT module was presented by a master’s-level registered nurse or a PhD-level cultural competency professional, or both, who were the lead trainers. The population-specific training modules were presented by a community member (a Marshallese trainer for the Marshallese CCT and a Hispanic trainer for the Hispanic CCT), who were supported by the lead trainers. The training modules included reference guides, handouts, information on cross-cultural communication tactics, interactive activities, a self-assessment of implicit bias, and a self-assessment of cultural competency. Each training module ended with a question-and-answer segment. Although organizations were encouraged to complete all 3 cultural competency training modules, the modules were offered separately, and organizations were allowed to choose the ones that best fit their needs.

The target audience was health care professionals, including physicians, nurses, pharmacists, health care educators, and allied health care professionals, as well as health care administrators and office staff members. Training sessions were held at the health care organization’s facility or a location convenient for the employees of each organization. An internal champion was identified at each organization, and that champion ensured that all staff members who had patient interaction were invited to participate. Most internal champions were a chief nursing officer or a director of human resources. All training sessions were offered free of charge and were certified for continuing education credits for physicians, pharmacists, nurses, and certified health care education specialists.

### Training evaluation and data collection

The training program was evaluated using a mixed-methods approach based on the Kirkpatrick Four-Level Training Evaluation Model (28). The Kirkpatrick model describes 4 levels of evaluation measures: 1) reaction, 2) learning, 3) behavior, and 4) results (28). Level 1 measures how attendees react to the training event (eg, satisfaction with the training, relevance to job duties). Level 2 measures what attendees learned or the extent to which knowledge was gained. Level 3 assesses whether the attendees changed any behaviors as a result of the training. Level 4 measures the degree to which targeted outcomes occurred as a result of the training. The evaluation of the training sessions focused on levels 2 and 3 (learning and behavior). We did not assess Level 1 measures as part of this evaluation, because this evaluation sought to understand what participants had learned and how participants and organizations changed behaviors on the basis of what they learned. The evaluation was unable to assess level 4 (the effects on patient care) at this time. Organizations are currently monitoring the long-term effects of the training program.

For individual participants, data on 5 items were captured by surveys at 2 points: immediately after each training session and 6 months after training. Surveys completed immediately after training collected basic information about participants (sex, occupation, whether English is primary language [yes/no], previous training in cultural competency [yes/no], and frequency of contact with other cultures [almost never/occasional/frequent]), and 3 yes/no survey items measured the trainings’ effects on knowledge, competence, and performance related to working with culturally diverse patients. These 3 items were designed according to the Accreditation Council for Continuing Medical Education’s Accreditation Criteria 2 and 11 (29). Participants indicated whether the training activities “increased [their] knowledge,” “increased [their] competence,” and/or “will improve [their] performance.” In addition, the immediate post-training surveys included open-ended questions on what the participants learned and how they planned to implement what they learned.

Six months after the training, participants completed a brief online follow-up questionnaire focused solely on behavior changes (level 3) to understand how participants implemented the information they learned (level 2). Among the questionnaire’s 3 open-ended questions was the following key question: “As a result of this cultural competency training program, did you personally make any specific changes in the way you do your job to improve the experience of patients/clients from a culture other than your own?” As an incentive to complete the 6-month follow-up questionnaire, participants who completed the follow-up questionnaire were entered into a drawing for a $50 gift card. Organizations were also contacted for an online interview 6 months after the training session to assess changes at the organizational level. One organizational representative was interviewed at each organization. This person was typically a chief nursing officer or a director of human resources. Organizational representatives were asked to report any organizational changes that occurred as a result of the training.

### Analysis

All quantitative and qualitative data analysis took place in February 2017. We examined immediate post-training survey results evaluating learning (level 2) and behavior (level 3). We conducted quantitative analysis based on the number of units of cultural competency training delivered, rather than the number of unique participants. Participants could be counted more than once if they completed an evaluation for more than one training module. Because all evaluation measures were completed anonymously, participants’ immediate post-training and 6-month follow-up responses could not be matched. We calculated descriptive statistics for all demographic items. For the 3 immediate post-training survey questions related to knowledge, competence, and performance, we calculated the percentage of responses that indicated increased knowledge, increased competence, or improved performance. We conducted qualitative analysis on more than 2,000 open-ended responses from the immediate post-training surveys, the 6-month follow-up questionnaires, and organizational interviews. We reviewed and coded responses by using the Kirkpatrick model’s learning (level 2) and behavior (level 3) domains as a priori codes. In the a priori codes, 3 qualitative themes emerged: 1) learning, awareness, and appreciation of cultural differences (level 2); 2) individual and organizational behavioral changes within human resources (level 3); and 3) individual and organizational behavioral changes in communication/materials (level 3). Two qualitative researchers (P.A.M., B.A.) independently coded the data and then compared and discussed the codes to ensure intercoder agreement. Three researchers (P.A.M., B.A., S.M.) then organized codes in a codebook and compared response themes from individual and organizational participants and found them to be consistent.

## Results

From March 19, 2015, to November 30, 2016, we conducted 51 in-person training sessions at 25 organizations and delivered 1,250 units of cultural competency training. Of those, 672 immediate post-training surveys were completed (response rate, 53.8%). The general CCT module was delivered to 370 health care professionals, the Hispanic CCT module was delivered to 382 health care professionals, and the Marshallese CCT module was delivered to 498 health care professionals. Most (88.1%) participants were women, and more than half (54.1%) had previous training in cultural competency. (Table 2).

**Table 2:** Characteristics of Participants (n = 672) in a Cultural Competency Training Program, Arkansas, 2015–2016^a^

Characteristic	No. (%)^b^
**Sex**	
Female	572 (88.1)
Male	77 (11.9)
**Occupation or profession **	
Administrative or human resources	127 (19.2)
Nursing	184 (27.8)
Physician	19 (2.9)
Social work	166 (25.0)
Other	167 (25.2)
**Is English your primary language? **	
Yes	636 (94.9)
No	34 (5.1)
**Have you ever had cultural competency training in the past? **	
Yes	360 (54.1)
No	306 (45.9)
**How much contact do you have with individuals from a culture other than your own?**	
Almost never	19 (2.9)
Occasional	217 (32.7)
Frequent	427 (64.4)

a Data were collected immediately after participation in the training session. Numbers reflect the number of evaluations, not the number of participants. Participants may have been counted more than once because they participated in multiple training sessions.

b Percentages are based on total number of valid responses to each item. Not all participants answered all questions. Percentages may not total 100 because of rounding.

Of the immediate post-training surveys with valid responses, 91.2% (602 of 660) reported an increase in knowledge, 86.6% (568 of 656) reported an increase in competence, and 87.2% (574 of 658) reported an improvement in performance. The percentage of participants who increased their knowledge (98.8%) and competence (96.1%) and improved their performance (94.6%) was greatest among participants in the Marshallese CCT module (Table 3).

**Table 3:** Results of Survey on Changes in Knowledge, Competence, and Performance, a Multicomponent Cultural Competency Training Program, Arkansas, 2015–2016

Module	No. of Respondents	No. (%)^a^
**General cultural competency training**		
Increased knowledge	177	149 (84.2)
Increased competence	176	138 (78.4)
Improved performance	178	146 (82.0)
**Hispanic cultural competency training**		
Increased knowledge	224	197 (87.9)
Increased competence	223	183 (82.1)
Improved performance	223	185 (83.0)
**Marshallese cultural competency training**		
Increased knowledge	259	256 (98.8)
Increased competence	257	247 (96.1)
Improved performance	257	243 (94.6)
**All modules**		
Increased knowledge	660	602 (91.2)
Increased competence	656	568 (86.6)
Improved performance	658	574 (87.2)

a Percentages are based on the number of valid responses to each item. Not all participants answered all questions.

### Qualitative results

Seventeen percent of participants responded to the 6-month follow-up survey. We also received feedback from 7 organizational representatives (response rate, 28.0%). Individual and organizational participants reported learning about cultural differences, as well as increased awareness and appreciation of the cultural differences of their patient population. Participants also reported behavior changes in human resources and in communication and materials.

**Learning, awareness, and appreciation of cultural differences.** Participants reported learning about the many elements associated with health and culture, with an emphasis on family, food, and health care practices in Hispanic and Marshallese patients. Individual participant A said, “I think about cultural significance of foods and of natural medicine and try to incorporate these things.” Individual participant A continued, “I try to keep [in mind] cultural practice and importance of cultural foods when talking about diet and explaining anatomy.” Other participants, including individual participant B, simply indicated they had become “more aware of family dynamics.”

Many participants, including individual participant C, indicated significant gains in cultural awareness and appreciation, reporting, “I became much more aware of how people within their own culture have differing perspectives based on their experiences.” Organizational participant A indicated that she has made an “attempt to be even more aware of cultural needs of the children and families we provide services to.” Individual participant A reported, “I try harder to be aware that other people may not have the same cultural upbringing as me. I try to watch for visual cues [like] body language or verbal tones to alert me of someone’s discomfort, and proceed accordingly.”

Similar changes to learning, awareness, and appreciation of cultural differences were reported at the organizational level. Organizational participant B reported “staff is much more aware of our Spanish and Marshallese patients and how to best interact with them.” Organizational participant C discussed the value of the training and how it continues to be used as a teaching method: “The knowledge I received from UAMS is very valuable when teaching others to be sensitive to the culture. By the end of this discussion the health care providers seem more understanding towards the Marshallese patients’ cultural differences.”

**Behavioral changes within human resources.** In addition to becoming more aware, participants reported changes to human resources at their workplaces. Human resources changes included recruitment efforts to create a more diverse applicant pool and hire more demographically diverse employees to better serve the diverse client population. Organizational participant A described human resources’ “increased efforts to recruit and hire Hispanic employees to better service our Hispanic families.” Organizational participant A’s organization has also “begun to brainstorm on how to increase efforts to recruit and hire bilingual employees to better provide services provided to our Marshallese families.” Organizational participant D stated, “I have worked with our HR person to get the Marshallese interpreter job description changed to better match the applicant pool.”

New roles and processes were also created to ensure that cultural competency trainings were incorporated into organizations and sustained. Organizational participant E stated, “For annual in-service, we created a short video in which we highlighted the challenges that come with seeking health care when major language barriers exist.” Organizational participant E also noted that “information on inclusion and cultural competency has been implemented in our new staff orientation.” Participants described new human resources practices, including the creation of a cultural champion. For instance, individual participant D’s organization “created a cultural competency champion for the organization. Added this team member to the ethics committee. Charged this team member to incorporate cultural learning opportunities with our team meetings” and will “provide ongoing team education.” Participants reported that staff meetings were being used as opportunities for cultural discussion. Individual participant E stated, “I have made it a point to discuss the cultural training material at staff meeting,” and noted that doing so allows for much broader dialogue about the need for culturally appropriate care.

**Behavioral changes in communications and materials.** Several participants made changes in their materials and communication methods to provide in-language oral and written communications to their patients and clients. One of the primary changes reported was having information translated into the primary language of the patients and having more interpreters available. Individual participant F described “utilizing medical interpreters for communication instead of relying on family members.” Participants also reported using teach-back communication methods suggested during the training. As individual participant G reported, “I make sure that I follow up more frequently and ask more specific questions during [health] assessment and/or evaluation. [I] try to have materials printed out in Marshallese for education.” Individual participant H explained they are continuing their efforts to obtain “more language specific materials,” and Individual participant I described efforts to get “more literature in different languages.” In addition, participating organizations stated they are investing in language services. Organizational participant E noted, “We have sponsored training for two Latino staff members in Spanish medical terminology.” Organizational participant B described how several changes are taking place simultaneously:

[The] training has increased awareness of various cultures throughout the facility and our clinics. For one, there is more signage in Marshallese and Spanish throughout the facility/clinics. We also plan to put up [bilingual] signage in our new women services facility. We also have more patient education in Marshallese and Spanish, and we are looking to having the rest of our materials translated.

Organizations also reported policies to ensure more culturally sensitive use of bilingual interpreters. For instance, organizational participant F stated, “I now require an interpreter of the same sex when possible for physicals and follow up for our Marshallese employees.”

## Discussion

Racial/ethnic minority populations encounter sociocultural barriers to quality health care, including implicit bias when seeking health care. Providing quality, culturally appropriate care to all community members is a priority of Healthy People 2020 (30). Although this evaluation was unable to capture and examine data on patient outcomes, results appear to be consistent with those found in several systematic reviews that illustrate the positive effects of cultural competency training programs on health care providers’ knowledge, competence, and skills for providing culturally competent care to diverse patient populations (10,16,17). Using a CBPR approach facilitated the creation of a unique community-driven cultural competency training program that was developed and delivered by Hispanic and Marshallese community members. This approach allowed the unique expertise of multiple partners to be incorporated into the trainings. Involvement from Hispanic and Marshallese community partners in the development and implementation of the training program allowed the communities’ most common concerns to be integrated into the trainings. Health care partners’ involvement ensured that trainings met their needs and addressed issues they experience in their practices. The continuing education credits were a benefit to providers. As an additional option, the training modules are being offered via the UAMS Learning on Demand online platform. These modules included the same content and were delivered by the same trainers as the in-person training modules. This offering has allowed health care providers to integrate the training modules into organizational development protocols and will help facilitate sustainability of the training series.

This evaluation went beyond traditional evaluations because it measured both knowledge and behavioral changes at the individual and organizational level by using a mixed-methods approach. The primary limitation of the evaluation is that it has yet to determine if the training modules will lead to long-term improvements in patient care. Additionally, the evaluation response rates were lower than expected, with only 53.8% of 1,250 training units evaluated, and only a 17.0% response rate to the 6-month follow-up questionnaires. The evaluation of changes in knowledge and behavior via self-report alone is also a limitation. However, substantial changes with examples were described in learning, awareness, and appreciation of cultural differences, as well as individual and organizational behavioral changes in human resources and communications and materials. The initial outcomes are encouraging and demonstrate the value of using a CBPR approach to develop and implement cultural competency training programs.
